# Oral self-injuries: Clinical findings in a series of 19 patients

**DOI:** 10.4317/medoral.19643

**Published:** 2014-12-05

**Authors:** Rosangela Cannavale, Angelo Itro, Giuseppina Campisi, Domenico Compilato, Giuseppe Colella

**Affiliations:** 1 DDS, Department of the Head and Neck Surgery. Second University of Naples, Naples. Italy; 2 MD, MDS, Department of the Head and Neck Surgery. Second University of Naples, Naples. Italy; 3 DDS,Department of Surgical and Oncological Disciplines, Section of Stomatological Sciences, Unit of Oral Medicine, University of Palermo. Italy

## Abstract

Objectives: Self-injury (SI) is defined as a behavioral disturbance consisting of a deliberate harm to one’s own body without suicidal intent, it is not uncommon and ranges in severity from simple nail-biting to more extreme forms of self-mutilation. The head neck region may be the target of such lesions. SI is associated with several medical conditions, of which it can represent the first clinical sign. Aim of this paper is to describe a series of oral SI, giving special emphasis to the clinical findings, etiology and the management of lesions. Material and Methods: A total of 19 patients with oral SI were prospectively examined; attention was paid to the occurrence and characterization of oral lesions. The management of the lesion also varied depending on the patient medical history, on the etiology of the psychiatric behavior, and on the severity, frequency, and method of inflicting injury. Periodic examinations were performed (after two weeks, three months and six months) and registered. Results: All the patients healed gradually and healing was conditioned by the disease underlying. The treatment consisted of behavior modification in 11 cases, pharmacological treatment in 11 cases, psychotherapy in 2 cases, mouth guard in 9 cases, surgery in 2 cases, extractions in 1 case. Conclusions: Oral SI are uncommon in the clinical practice. They may be associated with a known disease or may be the consequence of this, but often they may be the first sign of a psychiatric disorder.

** Key words:**Oral self-mutilation, self-inflicted lesions, self-injurious behavior, ulcers, Obsessive-Compulsive Disorder, mental retardation.

## Introduction

The terms self-injury (SI) or self-mutilation (SM) describes a form of behavioral disorder characterised by an intentional damage to a part of the body without a conscious purpose to commit suicide (1,2). To be considered as SI, a lesion must satisfy the following features: it must be socially unacceptable, repetitive and causing a mild or moderate tissue damage (3).

Although the prevalence of SI has not been clearly reported, it appears to be growing markedly in the last 20 years, passing from affecting 400 per 100 000 population in the early 1980s (4) to 750 per 100 000 in the late 1980s (5), and to 1000 per 100 000 in the late 1990s (6). Recently, Van Sell *et al*. have estimated that 3 million people in the United States present SI behaviors (7); however, because such behaviors are not considered socially acceptable and therefore usually hidden from others (8), the exact prevalence of self-mutilation in the world is uncertain and probably underestimated (9).

Furthermore, other most recent studies have reported much higher percentages depending on the different groups analysed. These data ranging from 4% in adults (3), to 17%-38% in university students (10,11), to 69% among high risk young people (substance abusers, victim of sexual abuse) (12), to 21%-82% among psychiatric patients (3,13) and to 7.7%-22.8% among institutionalized patients with mental retardation (14). As regards the prevalence of SI oral lesions in elderly, to date no epidemiological studies have been published on this topic. This suggests a low prevalence in this group although degenerative disorders (e.g. Parkinsonism and Alzheimer’s disease) are the most common conditions associated to oral SI (2).

The SI lesions are mainly caused by self-cutting, self-burning, self-scratching, self-biting, head-banging, self-hitting, SI tattoos, hair pulling, ingesting scrap, SI piercing, by the use of foreign bodies that cause wounds and by any action interfering with wound healing (e.g. excoriation of wounds) (2,5,15,16). From an anatomical point of view SI lesions are frequently located in the limbs and in the head and neck district, particularly in oral and peri-oral regions (17).

Often the arms are selected because their easy accessibility whilst the wrist and forearm may also be involved because “cutting your wrist” has long had connotations of suicide (3).

About 75% of SI lesions are located in the head and neck region (3). Oral structures such as the gum, the buccal mucosa, the tongue, the periodontal tissues and/or the teeth may be the targets of the SM. These oral lesions have been defined as factitial (i.e. factitial gingivitis, periodontitis or ulcer) (18,19) or SMs up to the tooth self-extraction (20). The wide range of the clinical presentation of the oral lesions, often not specific and mimicking other diseases could make the diagnosis a challenge for the clinicians, especially when the lesions are the first or the unique signs of unrecognized diseases.

Oral SMs can be classified as either organic or functional (21). In organic oral SM, the subjects are affected by genetic syndromes such as Lesch-Nyhan, Cornelia de Lange, Gilles de la Tourette, Munchhausen, Familial Disautonomia, congenital insensitivity to pain, and other entities such as autism, mental retardation, hereditary sensory neuropathies, encephalitis, congenital malformations, congenital infectious diseases, and epilepsy (18,19). In these patients, the injuries are inflicted unconsciously, in a compulsive form without specific intent (2). Conversely, in functional SI the lesions are caused consciously, as a response to certain stimuli, in order to attract the attention.

Although easy to apply, this classification could be considered obsolete and today, according to Simeon and Favazza (10) the SI behaviors are subdivided in four categories (stereotypic, major, compulsive and impulsive).

The management of oral SI behavior depends on the medical history of the patient, the etiology of the behaviors, the severity, frequency, and method of inflicting injury. On the basis of these considerations it is possible to opt for one or more of the following therapeutic modalities: psychological, pharmacological, conservative (use of intraoral device) and surgical.

The aim of this paper is to describe a series of oral SI injuries, giving special emphasis to the clinical findings, to the etiology and to the management of the lesions.

## Material and Methods

A series of 19 patients (11 males and 8 females), aged between 6 and 86 years ( SD ±23,53) , with oral SI were observed and treated from January 2001 to January 2013 at the Department of Head and Neck Surgery of the Second University of Naples ( Table 1). For each patient medical history and clinical findings were registered.

**Table 1:**
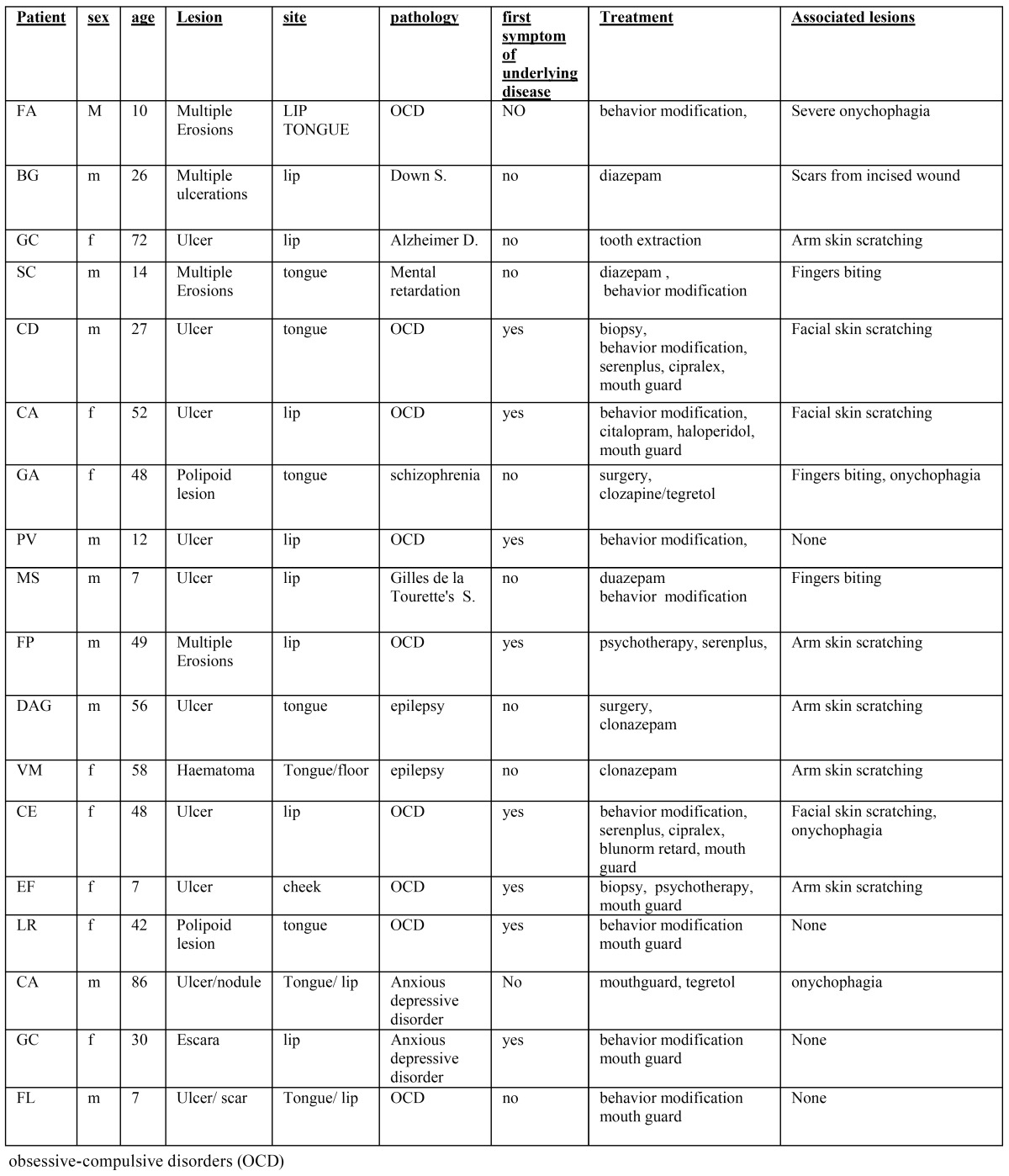
Demographic characteristics of included patients, pathology of patients and treatment.

Therapy was planned on the basis of the characteristics of the lesions (i.e. severity, site, modality of trauma), underlying illness, and psychiatric evaluation.

Periodic examinations were performed after the first two weeks (performed within 15 days from the diagnosis) to monitor SI. Follow-up period ranges from 3 months up to 6 months.

This study was approved by the ethics committee of Azienda Universitaria Policlinico of the Second University of Naples. We explained the details of this study to the patients in a document, and their written informed consent was obtained.

## Results

Oral lesions were localized mainly on the lips (8 cases out of 19), tongue (5 cases out of 19), lip and tongue (3 cases out of 19), in one case on oral floor and tongue, and in two cases on the cheek.

The intraoral examination revealed mainly: ulcerations (12 cases), multiple ulcerations (1 case), multiple erosions (3 cases), polipoid lesions (2 cases) and haematoma (1 case). Nine patients presented obsessive-compulsive disorders(OCD), 2 anxious-depressive disorders, 2 epilepsy, 1 Down’s syndrome, 1 Alzheimer disorder, 1 mental retardation, 1 schizophrenia, 1 Gilles de le Tourette, 1 psycho-affective immaturity.

Biopsy was performed in 2 cases due to a suspect of carcinoma (2 cases, both resulted negative).

Patients needed a multidisciplinary approach: behavioral modification techniques, pharmacological therapy and oral protections (i.e. mouth guard).

The treatment consisted of behavior modification in 11 cases, pharmacological treatment in 11 cases, psychotherapy in 2 cases, mouth guard in 9 cases, surgery in 2 cases, extractions in 1 case.

At follow-up, two weeks later, waiting for the healing of the injured tissues, a significant reduction in injury to the oral tissues was observed in the case of Alzheimer‘s disease (managed with tooth extraction to avoid further mucosal injuries). Also in 1 case of mental retardation managed with behavior therapy and pharmacotherapy (Diazepam), in schizophrenia managed with surgical and pharmacological approach (Clozapine/Tegretol) and in 2 cases of epilepsy managed with pharmacological therapy (Diazepam) in a case, plus suture of the wound in the second case the intraoral examination revealed a complete healing of the lesions.

In Gilles de la Tourette syndrome and Down syndrome no significant reduction of lesions were observed, multiple sections or ulcer were still observed on the lip because they continued to inflict trauma to the oral tissues. At follow-up, it has persisted lip ulcerations and lips swelling.

At three months follow-up in the cases of Alzheimer’s disease and schizophrenia, lesions healed completely.

The pharmacological therapy of epilepsy prevented the onset of new self-mutilation and allowed the total restitutio ad integrum of the lesions.

In Down syndrome the intraoral examination still did not shows improvements, even a number of new oral sites were observed, caused by sharp objects: at this time the traumatic lesions were on the ventral and dorsal sides of the tip of the tongue and on the lower lip mucosa and were painful to the touch.

At six months follow-up no signs of recurrence was observed in all cases, only in Down syndrome case, new extra-oral sites of injuries related to SI were observed: the tip of the fingers and the nails, resulting in traumatic amputations of the distal phalanx (parents reported cyclic psychiatric hospitalizations).

**Figure 1:**
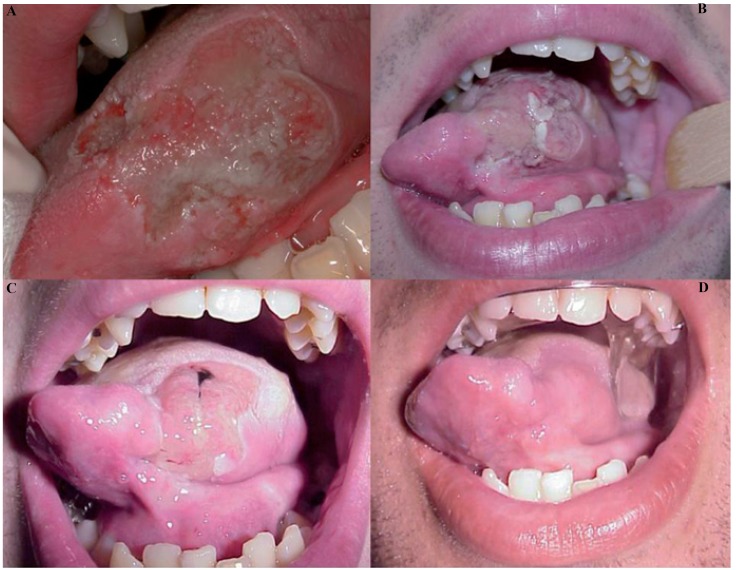
Fig. 1. OCD: Self- inflicted bite lesion on left side of the tongue: A) First observation. B) Worsening after one week. C) Initial repair of the lesion. D) Oral appliance with lingual shield, healed tongue showing loss of tissue.

In OCD patients and in two case of the anxious depressive disorder, managed by means of pharmacological therapy (Serenplus, Cipralex, Blunorm), behavioral modifications and a mouth guard, the intraoral examination two weeks after wearing the oral device, showed a significant reduction in injury to the oral tissues, then totally disappeared. The patients well tolerated the device; afterwards modifications of the design were performed on the basis of stability and retention to assure the patients compliance (Fig. 1).

In 2 cases of OCD managed by means of behavioral therapy used alone, the intraoral examination did not showed significant improvement of the lesions, also in 1 case of OCD managed by means of psychotherapy and pharmacological therapy no signs of healing or amelioration were observed.

At three months follow-up in OCD patients and in anxious depressive disorder case managed with mouth guard and pharmacological therapy and behavioral modification no further signs of injury were observed; in a 7 year old girl we observed the emergence of a similar lesion in the contra lateral cheek.

In one case of OCD (49 year old with multiple erosions on lip), lesions persisted likely related to the poor compliance with behavioral and pharmacological therapy. The patient was referred again to the psychiatric consultation. At six months follow-up, no occurrence of these lesions was observed in all patients.

## Discussion

Over the years a number of classifications of SI have been proposed (22-24). The most widely accepted has come from Simeon and Favazza (25,26). They propose that self-injurious behavior can be organized in four categories: stereotypic, major, compulsive, and impulsive. The “stereotypic” category refers to behaviors such as head banging, self-hitting, biting, picking, and scratching as enacted by individuals with mental retardation, developmental disability, autism, and other conditions (25). The “major SI” category (25) includes acts of self-harm that are often associated with psychosis and which cause considerable damage (self-enucleation, auto castration, self-amputation).

Compulsive SI refers to behaviors such as hair pulling, skin picking, and nail biting. Favazza (26) state that this category is associated with disorders such a trichotillomania and stereotypic movement disorder. These behaviors often occur several times per day and appears to have a strong association with the diagnoses of obsessive-compulsive disorder (OCD) (27). Simeon and Favazza (25,26) group SI skin cutting, burning, and hitting under the rubric of impulsive SI; these behaviors are associated with borderline personality disorder, antisocial personality disorder, post traumatic stress disorder (PTSD), and eating disorders.

SI has been considered resulting by five interrelated dimensions (28): environmental, biological, cognitive, affective, behavioral. The mix of dimensions is unique for each individual. The environmental dimension includes family historical elements (e.g. history of the family that have been “observed” but not directly experienced, i.e. to observe violence or substance abuse, violence, suicide, and self-injury in the family), patient historical elements (elements that have been “directly” experienced, i.e. death of a parent or other caregivers, loss through separation, divorce or placement outside the home, and experiences of neglect and/or emotional, physical and sexual abuse), and current environmental elements (circumstances in the present that tend to trigger SI, i.e. loss or conflict in relationships, abuse by a present caregiver or partner, exposition to peers who self-injure). (3,6,10,24,26,29-34)

The biological perspective of SI has changed with the increasing advances in brain imaging studies, abandoning the distinction between so-called “organic” and “functional” disorders, now considered obsolete. This distinction differentiated not disorders that were fundamentally different but only those in which we could measure a difference in the brain from those in which we could not. A number of psychiatric diagnoses associated with SI have been shown to have biochemical components, including borderline personality disorder, depression, bipolar illness, and schizophrenia (25). Other physiological problems commonly associated with SI include physical illness (e.g., diabetes, asthma, orthopedic disease), sleep disorder, eating disorder, and a tendency to somaticize distress.

The cognitive dimension falls into two basic categories: cognitive interpretations of environmental events and self-generated cognitions. Environmental events are problematic only if the self-injuring person interprets them to be aversive, painful, or disorganizing. Self-generated cognitions are triggered by internal cues as opposed to external events and circumstances. In addition, self-injuring persons generate a wide range of cognitions that trigger their acts of self-harm. Identifying these thoughts is another key step in assessment.

Closely linked to the cognitive dimension is the affective dimension. Emotions are often centrally important in assessing and treating SI. Most individuals self-injure in order to reduce or eliminate affective distress. Self-injuring persons identify a wide range of emotions as preceding their acts of self-harm, including anger, anxiety, tension, sadness, depression, shame, worry, and contempt (24,25,32,35,36).

The behavioral dimension consists of actions that immediately precede, accompany, and follow SI. These are strongly and recurrently associated with the acts of self-harm. Typical behavioral antecedents include conflicts with family or peers, isolation, failure at an activity, sexual behavior, substance abuse, or eating-disordered behavior. Also includes actions that prepare for SI, such as choosing the physical location, securing it to prevent interruption, and selecting a tool and actions that immediately follow SI, such as deciding whether or not to provide self-care, disposing of or hiding tools, and communicating with others. The aftermath of SI is very important to assess. Some individuals report falling asleep immediately after self-injuring; others return to normal activities; some remain agitated and seek other forms of release.

On this basis, this study intends to underline the difficulties in diagnosis the oral self-inflicted lesions.

A review of the literature (17) reveals that oral self-inflicted lesions are not so common but known since the late 1950s; there have been more than 55 cases published, within these the most extreme examples are auto-tooth extraction (37) and a mandible fractured. In the present study, we report a series of 18 consecutive patients observed and treated at our department, showing only lesions of the soft tissues.

To the best of our knowledge, there have been no previous reports presenting a so large series.

Although the typical clinical features of oral self-inflicted lesions are well documented, they often represent a challenge for the clinician and, even when recognized, their management are not well defined. There have been few reports of oral self-inflicted lesions being mistaken for more serious medical conditions (38); in fact in two cases a biopsy was performed to rule out any suspicion of malignancy.

Barrett *et al*. (39) reported a case that was initially mistaken clinically for an oral vesiculobullous disease, however, histopathology, direct immunofluorescence, and serology were all found to be negative. Other reports have described findings highly suspicious for malignancy, with one initially considered to be metastatic liposarcoma and another mistaken histopathologically for either leukemia or lymphoma, only ruled out after multiple tissue biopsies, molecular studies and bone marrow biopsy (38).

These reports stress the importance of obtaining a detailed clinical and social history, careful interpretation of laboratory results, and consideration of factitious disease in the differential diagnosis of cases that either present atypically, have a poor or incomplete history, or that do not respond to appropriate therapy (40).

In our observation, biopsy was performed in 2 cases due to a suspect of carcinoma (2 cases, both resulted negative) and one spinal tap (resulted negative) in order to exclude the choreo-acanthocytosis.

In our experience, patients with self-inflicted habits show a wider variety of types of injuries, equally prevalent in different age, in some cases obvious consequence of the underlying disease (i.e. wound and haematoma during epileptic fit). Extraction of the teeth becomes necessary to protect the patient from further injury (as in Alzheimer’s disease). Our clinical observations showed a notable frequency of lesions within OCD patients, mainly ulcerated lesions, representing the first sign of the underlying OCD. Sometimes is not easy to propose to these patients the psychological or psychiatric consultation, although this is essential to the success of treatment. According to Shaffer *et al*. these patients often have a borderline personality disorder and a stressful diathesis may be detected (41).

There are no standard techniques to prevent or treat oro-facial self-inflicted injuries. The treatment plan is established according to the special circumstances of the individual case.

A review of literature revealed common indicators that have been found to be associated with repeated self-harm in adolescents; these include personality disturbances, depression, alcohol and drug use, troubled relationships with peers and/or family members, poor school performance, and chronic psychosocial and behavioral problems (42).

Notably, in our series, OCD in children was related in all cases to troubled relationships with family members or conflicts between parents.

In general, the management of psychiatric forms was more challenging; after diagnosis, oral therapy focuses on symptomatic treatment to minimize tissue damage caused by SI, but addressing the underlying impetus for the behavior is essential for successful treatment, that involve a pharmacological and behavioral therapy. Symptomatic treatment, intended to protect oral tissues, included the use of a mouth guard, although it expects the patient’s compliance.

Finally, is important for clinicians to be aware of the existence of self-harm to identify them; to establish a dialogue with patients trying to overcome their distrust, in particular, in the case of pediatric patients, it is necessary to overcome the reluctance of parents, very often already aware of the problems of children.

## Conclusions

Oral SI is not frequently observed in oral medicine and surgery. Injuries caused by SI can be linked to various clinical conditions and can represent a diagnostic challenge, overall when they are the first or unique sign of an underlying disease. On other hand, in case of oral ulceration without apparent cause it is mandatory to rule out any other disease or malignancy before diagnosing a functional form. Successful treatment must be interdisciplinary and requires cooperation of patient, parents, health care providers, and medical team.

## References

[1] Favazza AR, DeRosear L, Conterio K (1989). Self-mutilation and eating disorders. Suicide & life-threatening behavior.

[2] Limeres J, Feijoo JF, Baluja F, Seoane JM, Diniz M, Diz P (2013). Oral self-injury: an update. Dent Traumatol.

[3] Briere J, Gil E (1998). Self-mutilation in clinical and general population samples: prevalence, correlates, and functions. The American journal of orthopsychiatry.

[4] Pattison EM, Kahan J (1983). The deliberate self-harm syndrome. Am J Psychiatry.

[5] Favazza AR, Conterio K (1988). The plight of chronic self-mutilators. Community Ment Health J.

[6] Favazza AR (1998). The coming of age of self-mutilation. J Nerv Ment Dis.

[7] Van Sell SL, O'Quin L, Oliphant E, Shull P, Johnston E, Nguyen C (2005). Help stop self-injury. Rn.

[8] Hicks KM, Hinck SM (2008). Concept analysis of self-mutilation. Journal of advanced nursing.

[9] Abraham G, Ilardi D (2005). Self mutilation: inward pain turned inside out. School Nurse News.

[10] Gratz KL, Conrad SD, Roemer L (2002). Risk factors for deliberate self-harm among college students. Am J Orthopsychiatry.

[11] Whitlock J, Eckenrode J, Silverman D (2006). Self-injurious behaviors in a college population. Pediatrics.

[12] Whitbeck LB, Chen X, Hoyt DR, Tyler KA, Johnson KD (2004). Mental disorder, subsistence strategies, and victimization among gay, lesbian, and bisexual homeless and runaway adolescents. Journal of sex research.

[13] Nock MK, Prinstein MJ (2004). A functional approach to the assessment of self-mutilative behavior. Journal of consulting and clinical psychology.

[14] Saemundsson SR, Roberts MW (1997). Oral self-injurious behavior in the developmentally disabled: review and a case. ASDC J Dent Child.

[15] Klonsky ED (2011). Non-suicidal self-injury in United States adults: prevalence, sociodemographics, topography and functions. Psychological medicine.

[16] Alderman N, Knight C, Morgan C (1997). Use of a modified version of the Overt Aggression Scale in the measurement and assessment of aggressive behaviours following brain injury. Brain Inj.

[17] Lucavechi T, Barberia E, Maroto M, Arenas M (2007). Self-injurious behavior in a patient with mental retardation: review of the literature and a case report. Quintessence Int.

[18] Medina AC, Sogbe R, Gomez-Rey AM, Mata M (2003). Factitial oral lesions in an autistic paediatric patient. Int J Paediatr Dent.

[19] Compilato D, Corsello G, Campisi G (2012). An unusual traumatic ulceration of the tongue. Journal of paediatrics and child health.

[20] Mass E, Gadoth N (1994). Oro-dental self-mutilation in familial dysautonomia. J Oral Pathol Med.

[21] Singh P, Emanuel R, Parry J, Anand PS (2008). Three paediatric patients with oral self-mutilation-a report. Dent Update.

[22] Menninger  KA (1963). Man against himself. Rupert HartDavis.

[23] Ross RR, McKay HB (1980). Self-mutilation. Lexington, Mass Heath; [Farnborough.
Faberow (Ed.). The Many Faces of Suicide..

[24] Walsh BW, Rosen PM (1988). Self-mutilation: theory, research, and treatment..

[25] Simeon D, Hollander E (2001). Self-injurious behaviors : assessment and treatment..

[26] Favazza AR (1996). Bodies under siege : self-mutilation and body modification in culture and psychiatry. 2nd ed. / Armando R. Favazza. ed..

[27] Christenson GA, Mackenzie TB, Mitchell JE (1991). Characteristics of 60 adult chronic hair pullers. Am J Psychiatry.

[28] Linehan M (1993). Cognitive-behavioral treatment of borderline personality disorder.

[29] Shapiro S, Dominiak GM (1992). Sexual trauma and psychopathology : clinical intervention with adult survivors. New York, N.Y: Lexington Books; Toronto: Maxwell Macmillan Canada.

[30] Miller D (1994). Women who hurt themselves: a book of hope and understanding.

[31] Van der Kolk BA, McFarlane AC, Weisæth L (1996). Traumatic stress : the effects of overwhelming experience on mind, body, and society.

[32] Alderman T (1997). The scarred soul: understanding & ending selfinflicted violence. Oakland, Calif: New Harbinger Publication: Distributed in the.

[33] Turell SC, Armsworth MW (2000). Differentiating incest survivors who self-mutilate. Child Abuse Negl.

[34] Paul T, Schroeter K, Dahme B, Nutzinger DO (2002). Self-injurious behavior in women with eating disorders. Am J Psychiatry.

[35] Favazza AR, Favazza B (1995). Bodies under siege : self-mutilation in culture and psychiatry.

[36] Conterio  K, Bloom JK, Lader W (1998). Bodily harm: the breakthrough treatment program for self-injurers. 1st pbk.ed..

[37] Altom RL, DiAngelis AJ (1989). Multiple autoextractions: oral self-mutilation reviewed. Oral Surg Oral Med Oral Pathol.

[38] Harrison M, Roberts GJ, Morgan PR, Pinkerton R (1998). Oral self-mutilation masquerading as malignancy. J R Soc Med.

[39] Barrett AP, Buckley DJ (1988). Covert self-mutilation of oral tissues and skin by mechanical and chemical means. Oral Surg Oral Med Oral Pathol.

[40] Fenton SJ (1982). Management of oral self-mutilation in neurologically impaired children. Spec Care Dentist.

[41] Shaffer M (1982). Life after stress.

[42] Wilkinson B (2011). Current trends in remediating adolescent self-injury: an integrative review. J Sch Nurs.

